# Coronary Microvascular Dysfunction Is Present Among Well-Treated Asymptomatic Persons With HIV and Similar to Those With Diabetes

**DOI:** 10.1093/ofid/ofae234

**Published:** 2024-04-26

**Authors:** Suman Srinivasa, Allie R Walpert, Daniel Huck, Teressa S Thomas, Carolyn N Dunderdale, Hang Lee, Marcelo F Dicarli, Gail K Adler, Steven K Grinspoon

**Affiliations:** Metabolism Unit, Massachusetts General Hospital and Harvard Medical School, Boston, Massachusetts, USA; Metabolism Unit, Massachusetts General Hospital and Harvard Medical School, Boston, Massachusetts, USA; Division of Nuclear Medicine and Molecular Imaging, Brigham and Women's Hospital and Harvard Medical School, Boston, Massachusetts, USA; Division of Cardiovascular Medicine, Brigham and Women’s Hospital and Harvard Medical School, Boston, Massachusetts, USA; Metabolism Unit, Massachusetts General Hospital and Harvard Medical School, Boston, Massachusetts, USA; Metabolism Unit, Massachusetts General Hospital and Harvard Medical School, Boston, Massachusetts, USA; Biostatistics Center, Massachusetts General Hospital and Harvard Medical School, Boston, Massachusetts, USA; Division of Nuclear Medicine and Molecular Imaging, Brigham and Women's Hospital and Harvard Medical School, Boston, Massachusetts, USA; Division of Endocrinology, Diabetes, and Hypertension, Brigham and Women’s Hospital and Harvard Medical School, Boston, Massachusetts, USA; Metabolism Unit, Massachusetts General Hospital and Harvard Medical School, Boston, Massachusetts, USA

**Keywords:** coronary flow reserve, HIV, myocardial blood flow, myocardial dysfunction

## Abstract

**Background:**

Coronary microvascular dysfunction (CMD) could be a potential underlying mechanism for myocardial disease in HIV.

**Methods:**

Comparisons of coronary flow reserve corrected for heart rate-blood pressure product (CFR_COR_) were made among people with HIV (PWH) with no known history of cardiovascular disease (CVD) or diabetes mellitus, persons without HIV (PWOH), and persons with diabetes (PWDM) and no known history of CVD or HIV.

**Results:**

PWH (n = 39, 74% male, age 55 [7] years, body mass index [BMI] 32.3 (26.8-34.9) kg/m^2^, duration of antiretroviral therapy 13 [5] years, CD4+ count 754 [598-961] cells/μL) were similar to PWOH (n = 69, 74% male, age 55 [8] years, BMI 32.2[25.6-36.5] kg/m^2^) and PWDM (n = 63, 63% male, age 55 [8] years, BMI 31.5 [28.6-35.6] kg/m^2^). CFR_COR_ was different among groups: PWOH 2.76 (2.37-3.36), PWH 2.47 (1.92-2.93), and PWDM 2.31 (1.98-2.84); overall *P* = .003. CFR_COR_ was reduced comparing PWH to PWOH (*P* = .04) and PWDM to PWOH (*P* = .007) but did not differ when comparing PWH to PWDM (*P* = .98). A total 31% of PWH had CFR_COR_ < 2.0, a critical cutoff for CMD, compared to 14% of PWOH and 27% with PWDM. A total 40% of women with HIV had a CFR_COR_ < 2.0 compared to 6% of women without HIV (*P* = .02).

**Conclusions:**

Subclinical CMD is present among chronically infected and well-treated, asymptomatic PWH who are immunologically controlled. This study demonstrates CFR is reduced in PWH compared to PWOH and comparable to PWDM, further highlighting that well-treated HIV infection is a CVD-risk enhancing factor for CMD similar to diabetes.

**Clinical Trials Registration:** NCT02740179

## Teaser

Reduced coronary flow reserve is present among asymptomatic, chronically infected PWH on ART who demonstrate good immunological control, similar to persons with DM. These data may suggest well-treated HIV infection is a CVD risk-enhancing factor similar to diabetes.

Persons with HIV (PWH) who are well-treated on antiretroviral therapy (ART) have an approximate 2-fold increased risk of cardiovascular disease (CVD) compared to persons without HIV (PWOH) [1, 2]. Although CVD is a critical leading comorbidity in PWH, little is known regarding the mechanisms of increased CVD risk in HIV.

A unique cardiovascular phenotype exists in HIV. Traditionally associated cardiovascular risk factors do not account for the excess CVD risk among PWH. To date, studies of CVD in HIV have largely been focused on atherosclerotic disease in the epicardial vessels, and little is known about disease of the coronary microvasculature. Flow-limiting impairments in the coronary microvasculature, including the prearteriole, arteriole, and capillary networks, cannot be captured on coronary computed tomography (CT) or invasive angiography and may otherwise be missed on diagnostic evaluation for coronary artery disease (CAD). Coronary flow reserve (CFR), the ratio of the maximal flow during vasodilator stress to the baseline flow at rest, can be assessed using positron emission tomography (PET) myocardial perfusion imaging and is a measure of the integrated hemodynamic effects of epicardial atherosclerosis and microcirculatory dysfunction on myocardial tissue perfusion [3]. In the absence of obstructive CAD, reduced CFR is a measure of coronary microvascular dysfunction (CMD) and has been associated with adverse cardiac events, including mortality [4, 5]. CMD may promote subendocardial ischemia and lead to subclinical cardiomyocyte injury, providing a potential underlying mechanism for the myocardial disease prevalent in HIV.

Only a few studies have assessed CFR in HIV [6], and none has compared CFR in PWH to a carefully defined population with metabolic disease and a well-matched control population. In the current study, we assessed CFR among those with HIV who were well-treated and at low risk for CVD by traditional indices, compared to a group of PWOH, referred for imaging with clinically available scans, and persons with diabetes (PWDM). The aim of the study was to assess the presence of CMD among a group of well-treated asymptomatic PWH without known CVD and to compare the degree of coronary microvascular dysfunction to a population with diabetes and no history of HIV, at higher risk for CVD, as well as to a population without diabetes or HIV.

## METHODS

### Participants With HIV

PWH were recruited as part of the MIRACLE HIV study (Mineralocorticoid Receptor Antagonism for Cardiovascular Health in HIV) conducted between February 2017 and March 2022 at the Massachusetts General Hospital and Brigham and Women's Hospital, which evaluated the use of mineralocorticoid receptor blockade in a randomized controlled trial on myocardial perfusion in HIV [7]. Per the study entry criteria, PWH aged 40 to 65 years on continuous antiretroviral therapy for >12 months with HIV viral load <100 copies/mL were included [7]. PWH were excluded for known history of CVD (CAD and heart failure), cerebrovascular disease, liver disease (alanine aminotransferase > 3 times the upper limit of normal) and kidney disease (creatinine > 1.5 mg/dL or estimated glomerular filtration rage [eGFR] < 60 mL/min/1.73 m^2^). For purposes of the current investigation, all available baseline CFR data were used, including from participants enrolled, but not randomized [7]. From this dataset, PWH with known diabetes mellitus (DM) or HbA1c ≥ 6.5% (n = 2) were excluded from the analyses to create the appropriate groups for comparison. Those on medications acting along the RAAS pathway (angiotensin-converting enzyme inhibitor, angiotensin II receptor blocker, mineralocorticoid receptor blockers) or diuretics or those with uncontrolled blood pressure (BP) > 140/90 mm Hg were excluded. Statin use was allowed if treatment was continuous for at least 12 months before CFR assessment. Women who were pregnant, seeking pregnancy, or breastfeeding were not permitted to participate. Any significant history of radiation within the preceding 12 months or evidence of coronary artery luminal narrowing >70% on coronary CT angiography was also exclusionary.

### Patient Consent Statement

All participants provided written informed consent to participate. This design of the work was approved by the Mass General Brigham Human Research Committee.

### Participants Without HIV

PWH were matched approximately 2:1 based on demographics (age ± 5 years, sex [male/female], race [White, Black, more than 1 race, other]), and traditional risk factors in sequential order of importance: BMI ± 1 kg/m^2^, history of hypertension (yes/no), history of tobacco use (yes/no), and history of dyslipidemia (yes/no) to individuals without HIV referred for clinical PET myocardial perfusion imaging to assess for CAD. Participants with obstructive CAD were ineligible for matching.

### Participants With DM

Baseline data from PWDM recruited as part of a previous randomized clinical trial evaluating effects of mineralocorticoid blockade on CFR in DM [8] were used a metabolic comparator group. In this study [8], PWDM were between the ages of 18 and 70 years and excluded for known CVD and cerebrovascular accident, renal disease (eGFR < 60 mL/min/1.73 m^2^), uncontrolled BP > 160/100 mm Hg, and current smoking.

### Cardiac PET

Study participants underwent cardiac PET myocardial perfusion imaging (whole-body PET/CT scanner Discovery RX or STE LightSpeed 64; GE Healthcare, Milwaukee, Wisconsin) after avoidance of caffeine, theophylline, or vasodilator substances for 24 hours. Calcium channel blockers were held for 36 hours. Regional myocardial blood flow was measured at rest and during vasodilator-stress with regadenoson (0.4 mg), dipyridamole (0.56 mg/kg), or adenosine infusion (140 mcg/kg/min) using nitrogen-13 (N-13) ammonia or rubidium-82 as a flow tracer. The flow tracer N-13 ammonia and the stress agent regadenoson were used for all PWH and PWDM. The flow tracer rubidium-82 (n = 55) and stress agent regadenoson (n = 55) was used in the majority of PWOH. Absolute myocardial blood flow (MBF) was computed from a 2-compartment tracer kinetic model using commercial software (Corridor4DM; Ann Arbor, Michigan) for rest and stress perfusion PET images, as described previously [9, 10]. A nongated, low-dose transmission CT scan was acquired for correction of photon attenuation. Heart rate, BP, and a 12-lead electrocardiogram were recorded at baseline and every minute after injection of vasodilator. Global CFR was determined as the ratio of peak MBF during vasodilator-stress to baseline flow and corrected for the baseline heart rate-BP product (RPP), using the formula CFR_corrected (COR)_ = CFR_uncorrected_ × RPP/10000 [10, 11]. Impaired CFR was defined as a ratio <2.0, and impaired stress MBF was defined as <1.8 mL/g myocardium/min based on prior studies [12]. Semiquantitative 17-segment assessment of myocardial perfusion images was performed by trained interpreters to assess summed rest and stress scores calculated from the sum of individual segment scores. In this scoring classification, summed stress scores of <4 indicate lower risk groups without evidence of obstructive disease. All quantitative analyses of PET images are motion corrected using the INVIA software. Rest left ventricular ejection fraction was also calculated from gated myocardial perfusion imaging. Quantitative or semiquantitative coronary artery calcium scores were determined from the Agatston score of gated CT scans or from visual assessment from the noncontrast CT scan obtained for attenuation correction as described previously [13].

### ASCVD Risk Score

The atherosclerotic cardiovascular disease (ASCVD) risk score was calculated using the pooled cohort equation, encompassing age, sex, race, total cholesterol, high-density lipoprotein, systolic BP and history of hypertension, DM, and tobacco use. For age, lipid, and blood pressure variables above or below the limits of the ASCVD risk calculator, the upper or lower boundary was used, respectively.

### Statistical Analysis

Normally distributed variables are reported as mean (SD), nonnormal distributed variables as median (interquartile range, 25th, 75th quartile), and categorical variables are shown as proportions. Overall group comparisons were performed using 1-way analysis of variance for normally distributed variables and the Kruskal-Wallis test for nonnormally distributed variables. If the overall group comparison were significant, then the 2 group comparisons of continuous variables were made using Student *t* test for normally distributed variables and Tukey-Kramer for nonnormally distributed variables. Additional analyses were performed after stratification of CFR using a cutoff of coronary flow reserve corrected for heart rate-blood pressure product (CFR_COR_) < 2.0, a value consistent with impaired microvascular flow. A chi-square test was used to determine whether there was a significant difference between the proportions. Determinants of CFR_COR_ were evaluated in multivariate regression modeling performed among the PWOH and PWH groups. Statistical significance was determined by a 2-sided *P* < .05. Analyses were performed using SAS JMP (Version 16).

## RESULTS

### Baseline Characteristics

Overall, PWH, PWOH, and PWDM were well-matched on age, sex, race, and ethnicity. The mean age of participants was 55 years. The majority of participants were of male sex, White, and non-Hispanic ethnicity (Table 1).

**Table 1.** Baseline Characteristics Among Groups

	Persons Without HIV/DM N = 69	Persons With HIV N = 39	Persons With DM N = 63	*P* Value
Demographics				
Age, y	55 (8)	55 (7)	55 (8)	.87
Male sex, %	74	74	63	.32
Race, %				.48
White	64	62	65	
Black	23	26	29	
Other	13	10	5	
Not reported	0	2	1	
Hispanic ethnicity, %	10	13	8	.35
Metabolic Risk Factors				
BMI, kg/m^2^	32.2 (25.6-36.5)	32.3 (26.8-34.9)	31.5 (28.6-35.6)	.77
Hypertension, %	54	23	57	.001
Tobacco use, %	10	23	0	<.0001
DM, %	0	0	100	<.0001
HbA1c, %	5.7 (5.5-6.1)	5.4 (5.2-5.9)	6.7 (6.2-7.2)	<.0001
Statin use, %	30	15	75	<.0001
Total cholesterol, mg/dL	178 (154-204)	182 (168-205)	149 (127-163)	<.0001
LDL, mg/dL	106 (74-121)	108 (77-127)	78 (64-94)	<.0001
HDL, mg/dL	53 (41-60)	46 (43-53)	42 (36-51)	.02
Triglycerides, mg/dL	129 (85-231)	159 (88-219)	101 (81-154)	.03
Cr, mg/dL	0.91 (0.19)	0.97 (0.14)	0.87 (0.15)	.007
eGFR, mL/min/1.73 m^2^	93 (81-102)	87 (76-93)	69 (60-96)	<.0001
ASCVD risk score	8.1 (4.1-13.7)	5.8 (3.4-8.9)	10.7 (4.7-20.8)	.005
ASCVD risk score >7.5%, %	53	33	62	.02
HIV Parameters				
CD4+ T-cell count, cells/μL	NA	754 (598-961)	NA	
Log HIV viral load, copies/mL	NA	1.34 (0.16)	NA	
Undetectable viral load, %	NA	85	NA	
Duration HIV (y)	NA	20 (8)	NA	
Duration ART use (y)	NA	13 (5)	NA	
Current PI use, %	NA	10	NA	
Current NRTI use, %	NA	100	NA	
Current NNRTI use, %	NA	49	NA	
Current integrase inhibitor use, %	NA	68	NA	

Data are represented as mean (SD) for normally distributed variables, median (interquartile range 25th-75th) for nonnormally distributed variables, or proportions.

Abbreviations: ART, antiretroviral therapy; ASCVD, atherosclerotic cardiovascular disease; BMI, body mass index; Cr, creatinine; DM, diabetes mellitus; eGFR, estimated glomerular filtration rate; HbA1c, hemoglobin A1c; HDL, high-density lipoprotein; LDL, low-density lipoprotein; NA, not available; NRTI, nucleoside/nucleotide reverse transcriptase inhibitors; NNRTI, nonnucleoside reverse transcriptase inhibitors; PI, protease inhibitor.

The median BMI for all groups was in the obese category. Fewer PWH had known hypertension compared to PWOH and PWDM. HbA1c was higher in the DM group as expected. Per protocol, PWH and PWOH were excluded for DM and demonstrated lower median HbA1c. PWH had higher triglyceride levels relative to PWOH and PWDM (Table 1). PWOH demonstrated higher resting systolic and resting diastolic BP compared to PWH. Resting heart rate did not differ by group (Table 2). Median ASCVD risk scores were lowest overall in the HIV group (5.8% [3.4%-8.9%]), with progressively higher levels among PWOH (8.1% [4.1%-13.7%]), and PWDM (10.7% [4.7%-20.8%]) (Table 1).

**Table 2.** Myocardial Blood Flow Assessed by Coronary PET Among Groups

	Persons Without HIV/DM N = 69	Persons With HIV N = 39	Persons With DM N = 63	Overall *P* Value
Resting Myocardial Blood Flow Parameters				
MBF, mL/min/g	0.81 (0.71-1.02)	0.65 (0.57-0.77)	0.69 (0.61-0.86)	<.0001^a,b^
HR, beats/min	68 (58-76)	68 (63-75)	67 (59-77)	.73
SBP, mm Hg	140 (128-156)	126 (114-139)	129 (120-144)	.0007^a,b^
DBP, mm Hg	78 (69-84)	70 (62-80)	74 (68-82)	.02^a^
RPP, beats/min x mm Hg	9577 (8382-10,829)	8448 (7680-9933)	8424 (7280-10,005)	.02
Stress Myocardial Blood Flow Parameters				
MBF, mL/min/g	2.51 (2.16-2.88)	1.80 (1.56-2.16)	1.92 (1.66-2.19)	<.0001^a,b^
HR, beats/min	90 (82-99)	92 (78-97)	92 (80-101)	.51
SBP, mm Hg	132 (116-149)	127 (116-138)	123 (112-139)	.02^b^
DBP, mm Hg	69 (61-79)	65 (60-72)	65 (61-75)	.13
Coronary Flow Reserve Parameters				
Summed stress score	0 (0-0)	0 (0-0)	0 (0-0)	.32
Global CFR_COR_	2.76 (2.37-3.36)	2.47 (1.92-2.93)	2.31 (1.98-2.84)	.003^a,b^
Other Myocardial Parameters				
LVEF, %	61 (56-66)	57 (52-59)	58 (53-64)	.01^a^
CAC severity, %				
Absent CAC	57	49	65	.77
Mild CAC 1-100	26	25	21	
Moderate CAC 101-300	10	17	9	
Severe CAC >300	7	9	5	

Overall group comparisons are made using the Kruskal-Wallis Test for nonnormally distributed variables. For significant overall group comparisons, paired comparisons of continuous variables are made using Tukey-Kramer for nonnormally distributed variables. All comparisons between person with HIV versus persons with DM were non-significant.

Abbreviations: CAC, coronary artery calcification; CFR_COR_, coronary flow reserve corrected for heart rate-blood pressure product; DBP, diastolic blood pressure; HR, heart rate; LVEF, left ventricular ejection fraction; MBF, myocardial blood flow; SBP, systolic blood pressure; RPP, heart rate-blood pressure product.

Significance between pairs is reported as follows.

^a^Persons without HIV/DM versus persons with HIV, *P* < .05.

^b^Persons without HIV/DM versus persons with DM, *P* < .05.

### Myocardial Blood Flow and Other Cardiac Parameters in Comparison to PWH

**Figure 1.**
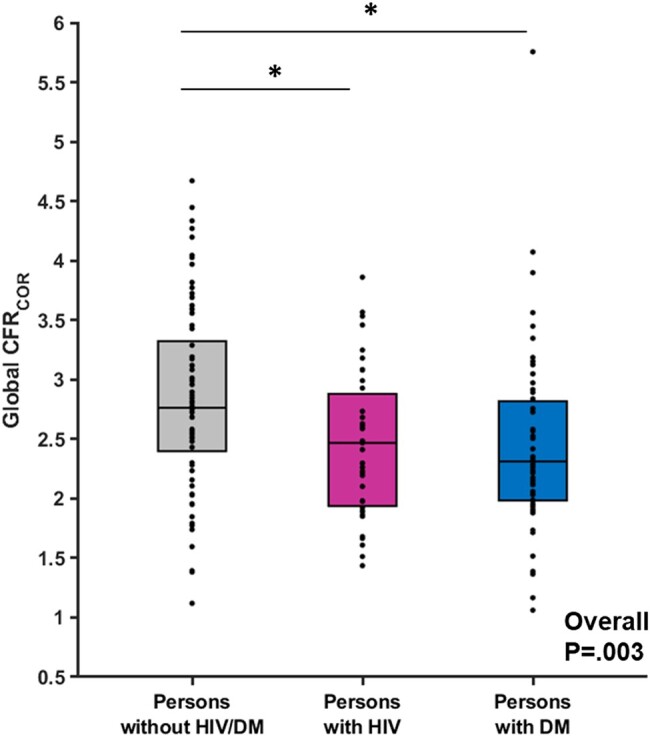
Coronary microvascular dysfunction among persons with HIV. Global CFR corrected for heart rate-blood pressure product (CFR_COR_) as assessed on coronary PET among persons without HIV/DM, persons with HIV, and persons with DM. Box plot represents the 25th and 75th percentile and line within the box represents the median. Dots represent data for each participant. Overall group comparison was made using the Kruskal-Wallis Test. Paired comparisons are made using Tukey-Kramer. *P < .05.

CFR_COR_ was reduced in PWH (2.47 [1.92-2.93]) and PWDM (2.31 [1.98-2.84]), compared to PWOH (2.76 [2.37-3.36]); overall *P* = .003 (Figure 1). CFR_COR_ did not differ among the PWH and PWDM groups (Table 2). A sensitivity analysis was performed excluding PWOH who did not receive regadenoson as the stress agent (n = 14). CFR_COR_ was 2.75 (2.23-3.28) among PWOH who received regadenoson. The main result remained unchanged in this sensitivity analysis (overall *P* = .02). The highest stress MBF was seen among PWOH, with similarly lower values for the PWH and PWDM groups (overall *P* < .0001) (Table 2). Differences in rest MBF were also seen among the groups (Table 2).

Overall, median left ventricular ejection fraction (LVEF) was preserved in all groups, though PWH demonstrated a relatively lower LVEF compared to PWOH (57% [52%-59%] versus 61% [56%-66]; *P* < .05). Only 2 participants had an LVEF < 40%, both of whom were PWDM. Coronary artery calcification (CAC) severity was not significantly different between the groups (Table 2). There were no significant differences in CFR in any of the 3 groups when stratified by absence and presence of CAC (CAC = 0 vs CAC > 0), *P* > .15 for all comparisons.

When stratified by CFR_COR_ < 2.0 to indicate CMD, there tended to be more participants meeting this criterion among PWH (31%) and PWDM (27%) compared to PWOH (14%), *P* = .09.

### Sex-specific Analysis of CFR

When stratified by CFR_COR_ < 2.0, there was a significantly higher proportion of women with HIV versus women without HIV and DM with impaired myocardial perfusion (40 vs 6%, *P* = .02).

### Multivariate Models to Assess Predictors of CFR Among PWH and PWOH

HIV serostatus was independently associated with decreased CFR_COR_ in models controlling for individual risk factors including current tobacco use, total cholesterol, triglycerides, statin use, eGFR, LVEF, or sex (all *P* ≤ .04 for HIV serostatus) (Table 3).

**Table 3.** Models to Assess Metabolic Variables as Determinants of Coronary Flow Reserve Among Persons Without HIV/DM and Persons With HIV

	Global CFR_COR_	
	Estimate (95% CI)	*P* Value
	(*R*^2^ = 0.06; *P* = .03)	
HIV serostatus	−0.1956	.01
Tobacco use	0.0910	.37
	(*R*^2^ = 0.07; *P* = .05)	
HIV serostatus	−0.1846	.02
Total cholesterol	0.0020	.34
	(*R*^2^ = 0.10; *P* = .01)	
HIV serostatus	−0.1872	.02
Triglycerides	0.0016	.06
	(*R*^2^ = 0.06; *P* = .04)	
HIV serostatus	−0.1907	.01
Statin use	−0.0458	.58
	(*R*^2^ = 0.05; *P* = .10)	
HIV serostatus	−0.1579	.04
eGFR	0.0010	.85
	(*R*^2^ = 0.06; *P* = .05)	
HIV serostatus	−0.1866	.02
LVEF	−0.0012	.88
	(*R*^2^ = 0.06; *P* = .05)	
HIV serostatus	−0.1838	.01
Male sex	−0.0125	.88

*R* ^2^ represents the coefficient of determination and the proportion of variance explained by the model. Overall *P* value represents significance by the whole model analysis of variance test. Abbreviations: CFR_COR_, coronary flow reserve corrected for heart rate-blood pressure product; eGFR, estimated glomerular filtration rate; LVEF, left ventricular ejection fraction.

## DISCUSSION

Our data show for the first time that CFR among PWH is reduced compared with PWOH and comparable to PWDM and further underscores well-treated HIV infection as a CVD risk-enhancing factor similar to diabetes. These data align with and expand those of the BETTER study to enhance our understanding of reduced CFR among PWH [6]. Prior studies have consistently shown that clinically relevant atherosclerotic disease or obstructive CAD are increased approximately 2-fold among PWH compared to PWOH [1, 2]. The current study importantly highlights that the spectrum of subclinical CVD in HIV should expand beyond the more well-recognized macrovascular complications to include significant microvascular disease, equivalent to that seen among a population with diabetes.

The median CFR_COR_ among PWH was almost 0.3 lower than PWOH. The observed difference in CFR may have clinical relevance because Kelshiker et al. has reported that a 0.1-unit reduction in CFR is associated with an 8% increase in the hazard of CV events [14]. Few other studies have evaluated CMD in HIV and have shown mixed results. Initiation of ART has been associated with a 20% reduction in myocardial flow reserve among PWH [15], though ART switch studies have shown some improvement among a subset of individuals with impaired CFR at baseline [6]. A prior study compared CMD in a group of PWH and PWOH and did not show a difference in CFR [16]. Another study by Knudesen et al. was similarly designed to compare CMD among PWH and PWOH with low Framingham Risk Score and low CAC burden; it showed reduced stress MBF among PWH but did not detect differences in CFR, in contrast to our study [17]. Importantly, we corrected for baseline differences in resting hemodynamics in this analysis to enable a comparison of CMD between groups.

Given some data to suggest alterations in microvascular flow in HIV, the implications of CMD in myocardial disease warrant further investigation in HIV. Alterations in myocardial perfusion could play a key role in the development of diastolic dysfunction and heart failure with preserved ejection fraction, both cardiovascular pathologies that are increased in prevalence among PWH [18–20]. In the general population, CMD is present in as many as 75% to 85% of those with heart failure with preserved ejection fraction [21, 22] and has been associated with diastolic dysfunction in low-risk patients with nonobstructive disease [23].

CFR is a useful marker of cardiovascular risk in the non-HIV population. In a cohort of patients referred for cardiac evaluation, it was demonstrated that CFR < 1.5 and CFR 1.5 to 2.0 had a hazard ratio of 5.6 and 3.4, respectively, for cardiac death when compared to patients with CFR >2.0 [24]. Moreover, cardiovascular mortality was increased among individuals without HIV with impaired versus normal flow reserve (4.2 vs 0.4%/y) [25]. In the current study, we stratified further by this critical cutoff. The proportion of PWH with impaired flow and a CFR_COR_ < 2.0 in this study was approximately double the proportion of PWOH. Moreover, none of the PWH had a CFR_COR_ > 4.0.

Overall, the groups in this study were carefully matched based on key demographic variables. In general, there was more tobacco use in the HIV population, which could contribute to CMD. After controlling for tobacco use, we were able to demonstrate that the reduction in CFR_COR_ was independent of tobacco use. Similarly, after taking into account total cholesterol, triglycerides, or statin use, HIV serostatus remained an important predictor of CMD. Despite lower traditional risk scores in PWH, compared to PWOH and PWDM, PWH had more CMD than PWOH and similar CMD to PWDM.

It is well-known that CVD risk calculators, such as the ASCVD risk score, underestimate the risk of CVD in HIV [26]. The ASCVD calculator using the pooled cohort equation recognizes DM as a risk-enhancing factor but does not formally account for HIV as a risk-enhancing factor. A recent American Heart Association scientific statement suggests a pragmatic approach in which providers caring for PWH should consider factors such as prolonged viremia, delay in ART initiation, low nadir or current CD4, hepatitis C virus coinfection, and metabolic indices, such as lipodystrophy, when assessing for ASCVD risk [27]. We studied a population of PWH with a long duration of HIV, well-controlled on ART with good immunologic control and low levels of viremia, which represents the large portion of PWH with access to care and demonstrate this population to have evidence of subclinical CMD. Additional studies are needed to investigate how variable presentations of HIV (ie, duration of HIV, duration of ART, and comorbidities of HIV) impact microvascular disease and may inform us further about predictors of CVD risk in HIV.

There was also a low proportion of participants with severe CAC, again suggesting a low prevalence of diffuse atherosclerosis in any of the groups. Impaired CFR has been shown to predict cardiac mortality independent of obstructive disease [4, 5]. Recent studies have also shown a link between CMD and major adverse cardiovascular events [28]. Although we may hypothesize that microvascular disease precedes macrovascular disease in HIV, further studies investigating the natural history of microvascular and macrovascular disease in HIV should be designed to understand this relationship further.

The study enrolled few female participants, but nonetheless begins to suggest sex-based differences. A high percentage of women with HIV met criteria for a CFR_COR_ < 2.0, which was approximately 6 to 7 times higher than the percentage of women without HIV with CFR_COR_ < 2.0. In contrast, the percentage of women with CFR_COR_ <2.0 was similar among PWH and PWDM. Within HIV itself, the CFR_COR_ appears lower among women when compared to men, but we were unable to detect a statistically significant difference because of smaller sample sizes in the sex-stratified models. Using rubidium-82 PET imaging, Knudsen et al. showed that 45% of women with HIV and very low CVD risk at baseline had a CFR < 2.0 [29], and our data similarly replicate this prevalence among a comparable well-treated, low-risk group with 40% of women with HIV demonstrating a CFR_COR_ < 2.0. Larger studies are needed to understand microvascular disease in women with HIV. The INFORM study (NCT04224181) is currently under way and is specifically aimed at investigating CFR among women with HIV versus women without HIV.

There were a few limitations with the study. Both N-13 ammonia and rubidium-82 were used as flow tracers but have demonstrated reproducibility and are comparable for assessing CFR [9]. A small portion of PWOH received a stress agent other than regadenoson, though on excluding these PWOH in a sensitivity analysis, differences among the 3 groups remained. Group comparisons were made from cohorts recruited for different purposes, either for clinical referral or for research protocols. As the PWOH were leveraged from a clinical cohort, we did not have access to other data that would have been collected as part of a research investigation, such as inflammatory markers, and were only able to draw comparisons based on the data available. Nonetheless, a strength of the study was to compare the PWH to a group of well-matched PWOH who were referred for clinical assessment of CVD. In addition, we were able to make a comparison of CFR measures in PWH to PWDM, another metabolic group known to be at high risk for developing CVD. Comparator groups were useful to place the CFR data obtained from PWH in clinical context. Moreover, we were able to measure microvascular flow using coronary PET, which is the gold standard technique to assess for CMD. CFR provides key information about the vasculature in the myocardium, whereas techniques, such as flow-mediated dilation, are limited to providing information about the peripheral vasculature.

Overall, our data show that subclinical coronary microvascular dysfunction is present among chronically infected and asymptomatic PWH on ART who are immunologically controlled. Interestingly, the degree of CMD is similar to PWDM and further emphasizes HIV as a CVD risk-enhancing factor, similar to DM. Early perturbations in microvascular flow may drive alterations in cardiac structure and function in HIV before clinical disease presents. Although there are studies in the general population to suggest reduced CFR is related to increased CV mortality, future studies are needed to fully understand the role of CMD in myocardial disease in HIV.

## Notes

This abstract was presented at The Endocrine Society Annual Conference in June 2022.

***Author Contributions.*** Concept and design: S. S., A. R. W., M. F. D., G. K. A., and S. K. G. Acquisition, analysis, or interpretation of data: all authors. Drafting of the manuscript: S. S., A. R. W., D. H., M. F. D., G. K. A., and S. K. G. Critical review of the manuscript: all authors.

***Financial support.*** Funding was provided by National Institutes of Health (NIH) R01 DK49302 to S. K. G. and G. K. A.; NIH K23 HL136262 and R01 HL151293 to S. S.; NIH T32 HL094301 and American Heart Association Career Development Award 23CDA1037589 to D. M. H.; NIH K24 HL103845 to G. K. A.; NIH P30 DK040561 to Nutrition and Obesity Research Center at Harvard; NIH 1UL1TR001102 and 1UL1TR002541 to Massachusetts General Hospital Translational and Clinical Research Centers. Funding sources had no role in the design of the study, data analysis, or writing of the manuscript.

***Data availability.*** Datasets generated during and/or analyzed during the current study are not publicly available but are available from the corresponding author on reasonable request.
